# Inhibitory Effect of Sodium Alginate Nanoemulsion Coating Containing Myrtle Essential Oil (*Myrtus communis* L.) on *Listeria monocytogenes* in Kasar Cheese

**DOI:** 10.3390/molecules27217298

**Published:** 2022-10-27

**Authors:** Gökçe Polat Yemiş, Elif Sezer, Hatice Sıçramaz

**Affiliations:** 1 Department of Food Engineering, Faculty of Engineering, Sakarya University, Serdivan 54187, Turkey; 2 Sakarya University Research, Development, and Application Center (SARGEM), Serdivan 54187, Turkey

**Keywords:** myrtle essential oil, alginate nanoemulsion coating, *L. monocytogenes*, Kasar cheese

## Abstract

The present study aimed to characterize the physical properties of nanoemulsion-based sodium alginate edible coatings containing myrtle (*Myrtus communis* L.) essential oil and to determine its inhibitory effects on *Listeria monocytogenes* in fresh Kasar cheese during the 24-day storage at 4 °C. The GC-MS analysis showed that the main components of myrtle essential oil were 1,8-cineol (38.64%), α-pinene (30.19%), d-limonene (7.51%), and α-ocimene (6.57%). Myrtle essential oil showed an inhibitory effect on all tested *L. monocytogenes* strains and this effect significantly increased after ultrasonication. Minimum inhibitory and minimum bactericidal concentrations of myrtle essential oil nanoemulsion were found to be 4.00–4.67 mg/mL and 5.00–7.33 mg/mL, respectively. The antibacterial activity of myrtle essential oil nanoemulsion against *L. monocytogenes* was confirmed by the membrane integrity and FESEM analyses. Nanoemulsion coatings containing myrtle essential oil showed antibacterial activity against *L. monocytogenes* with no adverse effects on the physicochemical properties of cheese samples. Nanoemulsion coatings containing 1.0% and 2.0% myrtle essential oil reduced the *L. monocytogenes* population in cheese during the storage by 0.42 and 0.88 log cfu/g, respectively. These results revealed that nanoemulsion-based alginate edible coatings containing myrtle essential oil have the potential to be used as a natural food preservative.

## 1. Introduction

*Listeria monocytogenes* is one of the most important foodborne pathogens that causes listeriosis in humans and animals. Soft and semi-hard cheeses are considered important risk products for foodborne listeriosis and were identified as the major food vehicle of *L. monocytogenes* infections. Control of *L. monocytogenes* in cheese processing is particularly difficult due to its high cold and osmotic stress tolerance, and its ability to form environmentally stable biofilms resistant to sanitation. *L. monocytogenes* contamination is associated with inadequately pasteurized milk and post-process contamination or inadequate processing [1,2]. Kasar cheese is a semi-hard cheese commercially produced in Turkey and constitutes a significant part of the annual total cheese consumption (242 thousand tons/year). Kasar cheese has an ideal environment for the growth of many microorganisms, including *L. monocytogenes* due to its high-water content (45%) and suitable pH (5.5–5.8) [3,4].

The microbiological safety of food is a major concern for consumers, regulatory agencies, researchers, and the food industry around the world. There is increasing interest in the use of natural or plant-based preservatives such as essential oils for maintaining food quality and safety. Essential oils and their constituents have great potential as natural antimicrobial agents to prevent the growth of pathogenic and spoilage microorganisms in foods. Essential oils are naturally aromatic and volatile liquids obtained from different parts of plants such as flowers, roots, bark, leaves, seeds, and fruits. It has been reported that essential oils exert potent antimicrobial, antiviral, antifungal, and antioxidant effects, depending on their active constituents [5,6,7]. Nevertheless, their poor solubility in water, susceptibility to oxidative degradation, high volatility, and intense aroma all limit their use in food products. These disadvantages of essential oils can be overcome by incorporating them into nanoemulsion-based delivery systems. Recently, researchers have focused on the use of bioactive compounds with natural antimicrobial properties in food preservation in nanoemulsion against pathogenic and spoilage microorganisms [8]. Ultrasonication has been considered an efficient and eco-friendly physical method for nanoemulsification. The reduction in particle size of emulsions by ultrasound treatment enhances the accessibility of bioactive agents and improves their antimicrobial properties. When the bioactive components are encapsulated with a suitable transport system, their interaction with other food components is prevented, the physical stability of the active substances is increased, and their controlled release into the food can be realized. Nanoemulsions have a key role in the development of a new generation of active food packaging. Bio-based packaging has been increasing in popularity given its beneficial impact on the environment. Sodium alginate is one of the most important natural biopolymers used in the preparation of biodegradable films and edible coatings. It has been reported that nanoemulsions containing active ingredients can be applied as edible coatings, which is a promising method to improve the quality and safety of foods [9,10,11].

Myrtle (*Myrtus communis* L.) belongs to the *Myrtaceae* family and is a characteristic plant representative of the Mediterranean flora. It has been reported that the chemical composition of myrtle essential oil consists mainly of α-pinene, limonene, 1,8-cineole, linalool, α-terpineol, linalyl acetate, α-terpineol acetate, and geranyl acetate. The antibacterial properties of myrtle essential oils against pathogenic bacteria were reported in many studies and obtained results are promising. However, there are only a few reports on the application of myrtle essential oil as an antimicrobial in foods for the control of *L. monocytogenes* [12,13]. The antibacterial activity of myrtle essential oil was associated with α-pinene, 1,8-cineole, and linalool [14,15,16]. The use of essential oils as natural preservatives in different types of cheese has been widely studied in recent years. On the other hand, despite the essential oils exhibiting promising antimicrobial and antioxidant activity, their use is still limited in the cheese industry due to their negative effect on the physicochemical and sensorial properties of the final products. To overcome these drawbacks, nanoemulsion-based edible coating applications in which essential oils are encapsulated could be a promising alternative approach. To the best of our knowledge, no studies on ultrasound-treated sodium alginate-based edible coating containing myrtle essential oil and the application of these coatings on food have been reported. The present study aimed to determine the antibacterial effect of sodium alginate nanoemulsion-based edible coatings containing different concentrations of myrtle essential oil produced by ultrasound treatment on *L. monocytogenes* in Kasar cheese. In addition, the effect was examined of the nanoemulsion coating on the physicochemical and sensory properties of cheese.

## 2. Results and Discussion

### 2.1. The Chemical Composition of Myrtle Essential Oil

Essential oils and their bioactive constituents play an important role in antimicrobial activity. Twenty-five components accounting for 97.86% of the total composition of myrtle essential oil were identified. The relative percentages of the components are presented in Table 1. The major components of myrtle essential oil were 1,8-cineol (38.64%), α-pinene (30.19%), d-limonene (7.51%), and α-ocimene (6.57%), whereas α-terpineol (3.91%), β-cis-ocimene (2.68%), isosylvestrene (2.2%), o-cymene (1.14%), and myrtenol (1.03%) were also present in relatively high amounts. Previous studies reported that the main constituents of myrtle essential oil were 1,8-cineol (5.92–32.12%), α-pinene (9.00–44.62%), d-limonene (trace–23.55%), linalool (2.07–29.08%), and α-terpineol (0.42–8.12%) [17,18,19,20]. The variability of the chemical composition of myrtle essential oil has been associated with geographic origin, variety, plant parts, season, extraction type, and storage conditions of the essential oil [16]. 

### 2.2. Antibacterial Efficiency of Myrtle Essential Oil Nanoemulsion

The antibacterial effect of myrtle essential oil emulsion and nanoemulsion against *L. monocytogenes* strains was determined quantitatively by the microtube dilution method, and the obtained minimum inhibition concentration (MIC) and minimum bactericidal concentration (MBC) values are given in Table 2. Myrtle essential oil emulsion and nanoemulsion showed an inhibitory effect on all tested *L. monocytogenes* strains. Monoterpene hydrocarbons and oxygenated monoterpenes, such as 1,8-cineol, α-pinene, linalool, α-terpineol, and γ-terpinene in the myrtle essential oil, are responsible for the antibacterial activity against Gram-positive and Gram-negative bacteria, mainly *Listeria* spp. [12,13,21,22]. Similarly, in the present study, the antimicrobial activity of myrtle essential oil was associated with the high content of 1,8-cineol (38.64%), and α-pinene (30.19%). In addition, it has been reported that antimicrobial activity was highly correlated with the synergetic effect between the major and minor compounds in myrtle essential oil, rather than a single compound [23]. The MIC and MBC values of myrtle essential oil for *L. monocytogenes* strains were found to be 6.00–8.67 mg/mL and 8.00–14.67 mg/mL, respectively (Table 2). The results revealed that *L. monocytogenes* ATCC 13932 was the most resistant strain in the group. There are limited studies in the literature examining the antimicrobial effect of myrtle essential oil on *L. monocytogenes*. Dhifi et al. [12] reported that the MIC and MBC values of myrtle essential oil for *L. monocytogenes* was 4 mg/mL and 8 mg/mL, respectively. Similarly, Akin et al. [21] and Caputo et al. [22] declared that the MIC value of myrtle essential oil was 5 mg/mL and 3 mg/mL, respectively. These differences can be explained by the chemical composition of the essential oil and the difference in the strains used. In contrast, myrtle essential oil nanoemulsion exhibited higher antimicrobial activity against all tested *L. monocytogenes* strains compared to that of the myrtle essential oil emulsion (*p* < 0.05). The MIC and MBC values of the myrtle essential oil nanoemulsion were determined to be 4.00–4.67 mg/mL and 5.00–7.33 mg/mL, respectively. The antibacterial effect significantly increased as a result of ultrasound treatment (*p* < 0.05). Similarly, Kazemeini et al. [24] have reported that the nanoemulsion of *Trachyspermum ammi* essential oil had lower MIC values against *L. monocytogenes* than its pure oil. Moghimi et al. [25] have reported that the antimicrobial activity of sage essential oil increased after its incorporation into the nanoemulsion. Thyme essential oil [26], *thymus daenensis* essential oil [27], and cinnamon essential oil [28] yielded similar results in studies on the antibacterial activity of nanoemulsions. In ultrasonic emulsification, ultrasonic waves generate cavitation forces that convert the macroemulsion into nanoemulsion. Nanoemulsions obtained by ultrasonication have higher stability and lower droplet size. The encapsulation of essential oil in nanoemulsion systems can enhance their antimicrobial activity by the reduction in the size of essential oil droplets in nanoemulsion. The increased surface area of antimicrobial compounds provides better interaction with the cell membrane leading to increasing antimicrobial activity.

**Figure 1:**
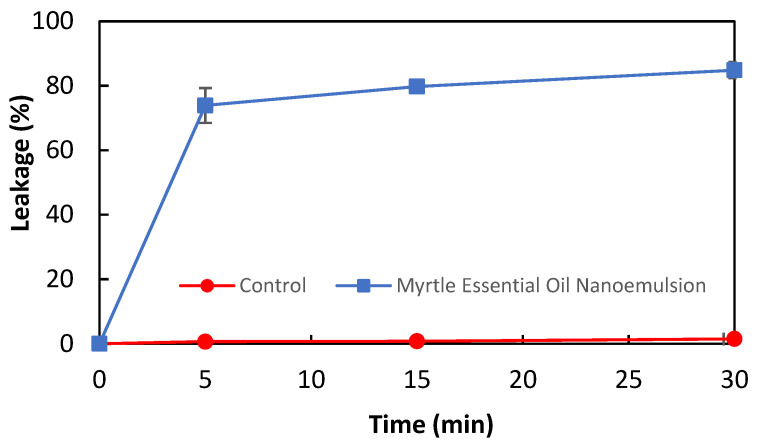
The effect of myrtle essential oil nanoemulsion on the leakage of nucleic acids of *L. monocytogenes* cells.

The antibacterial activity of myrtle essential oil nanoemulsion against *L. monocytogenes* was confirmed by the membrane integrity analysis. The release of nucleic acids from bacterial cells is considered an indicator of a decrease in cell membrane integrity. The effect of myrtle essential oil nanoemulsion on the membrane integrity of *L. monocytogenes* is shown in Figure 1. The nucleic acid leakage in bacterial cells treated and untreated with myrtle essential oil nanoemulsion was 73.88% and 0.64% at the 5th minute, and 84.85% and 1.5% at the 30th minute, respectively. These results revealed that the myrtle essential oil nanoemulsion damaged the cell membrane of *L. monocytogenes* and caused the intracellular components to leak out in a short time. It has been reported that essential oils damage the cell membrane of bacteria and then lead to the leakage of intracellular contents such as nucleic acids and protein [29,30]. Sikkema et al. [31] have reported that the cyclic terpene hydrocarbons such as α-pinene, β-pinene, γ-terpinene, and limonene affect the structure and function of the bacterial cell membranes.

**Figure 2:**
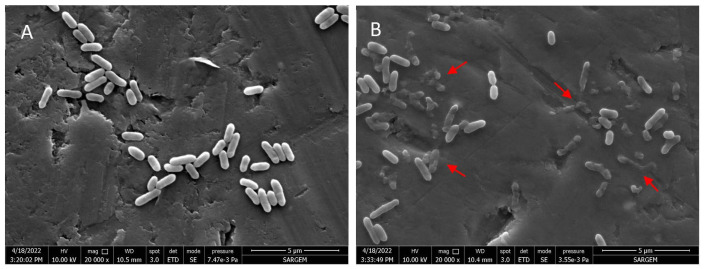
Scanning electron micrographs of cell morphology of *L. monocytogenes* untreated (**A**), treated with nanoemulsion of myrtle essential oil at MBC for 15 min (**B**).

The effect of myrtle essential oil nanoemulsion on the cell morphology of *L. monocytogenes* was investigated by Field Emission Electron Microscopy (FESEM). The cells treated or untreated with myrtle essential oil nanoemulsion are shown in Figure 2. The untreated *L. monocytogenes* cells exhibited rod-shaped structures with intact cell integrity and smooth and robust surfaces, while a wide variety of structural disruptions with varying degrees of shrinkage and destruction were observed in bacterial cells treated with myrtle essential oil nanoemulsion. The FESEM images showed that the myrtle essential oil nanoemulsion caused significant damage to bacterial cell integrity.

### 2.3. Characterization of Emulsions and Nanoemulsions Coating

The mean particle size, zeta potential, and whiteness index (WI) values of alginate-based emulsions and nanoemulsions containing myrtle essential oil are shown in Table 3. Nanoemulsions with smaller particle sizes were obtained by the ultrasound process compared to those of coarse emulsions. The particle sizes of nanoemulsions containing 0.5%, 1.0%, and 2.0% myrtle essential oil were 157 ± 22 nm, 172 ± 30 nm, and 122.7 ± 1.2 nm, respectively. The results were similar to those reported by Artiga-Artigas et al. [32] who studied alginate-based nanoemulsions containing different concentrations of oregano essential oil and mandarin fiber. Similar particle size results for nanoemulsions produced by ultrasound have been also reported by Rahmasari and Polat Yemiş [33], Salvia-Trujillo et al. [34], and Chu et al. [35].

The ζ-potential is a key parameter for evaluating the stability of the colloidal system. The particle with a ζ-potential value more positive than +30 mV or more negative than −30 mV represents appropriate stability of the emulsion. All nanoemulsions obtained by ultrasound treatment showed negative ζ-potential values of lower than −30 mV and a more stable structure (−38.47 ± 2.48, −32.27 ± 2.94, and −37.37 ± 2.48 for nanoemulsions containing 0.5%, 1.0%, and 2.0% myrtle essential oil, respectively). There were no significant differences between the formulations (*p* > 0.05). The coarse emulsions, on the other hand, exhibited a weak surface charge with negative values of lower than −30 mV. The nanoemulsions obtained in the study showed higher stability compared to the coarse emulsions. It has been reported that several mechanical stresses such as the micro-fluidization and ultrasound process can lead to the release of free carboxyl and hydroxyl groups, which are responsible for the negative charge of the essential oil nanoemulsion [11,36].

The optical properties of nanoemulsions are an important factor in food applications. The whiteness index (WI) of emulsions containing different concentrations of myrtle essential oil decreased significantly (*p* < 0.05) after the ultrasound treatment. Similarly, Salvia-Trujillo et al. [37] have reported a decrease in WI values of alginate-based nanoemulsions containing essential oil after the micro-fluidization process. The whiteness index is closely related to the particle size of the nanoemulsions. It has been reported that larger particles scatter light more intensely than small particles, causing an increase in the whiteness index of emulsions [38]. However, the WI values of the obtained emulsions and nanoemulsions decreased due to the increase in essential oil concentration (*p* < 0.05). It has been reported that the color of the emulsion depends on the scattered light apart from the droplet size, the refractive index of the continuous and dispersed phase, and the oil concentration [39].

### 2.4. Application of Nanoemulsion Edible Coatings on Cheese Samples

#### 2.4.1. Antibacterial Activity against *L. monocytogenes* in Cheese Samples

**Figure 3:**
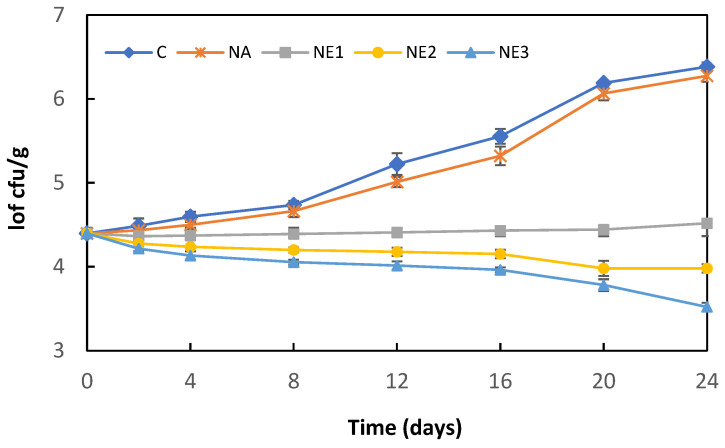
Changes in *L. monocytogenes* counts of cheese samples during storage. C: Control, uncoated cheese; NA: samples coated ultrasound-treated sodium alginate solution without the incorporation of myrtle essential oil; NE_1_, NE_2_, NE_3_: samples coated ultrasound-treated sodium alginate solution with the incorporation of 0.5, 1.0, or 2.0% myrtle essential oil, respectively. Error bars represent standard deviation.

The effect of sodium alginate nanoemulsion coatings containing different concentrations of myrtle essential oil on the *L. monocytogenes* counts in fresh Kasar cheese during storage at 4 °C is shown in Figure 3. The initial *L. monocytogenes* count (4.40 log cfu/g) increased significantly after 24 days of storage in both control (C) and alginate nanoemulsion coating (NA)-samples, reaching 6.38 and 6.27 log cfu/g, respectively (*p* < 0.05). The *L. monocytogenes* counts in the C and NA samples were higher than those of the alginate nanoemulsion-coating samples containing myrtle essential oil (NA_1_, NA_2_, and NA_3_) during the storage period. The growth of *L. monocytogenes* was suppressed in the coating samples (NE_1_) containing 0.5% myrtle essential oil; however, no significant changes were observed in the bacterial counts during the storage (*p* > 0.05). Similarly, previous studies have reported myrtle essential oil has a lower anti-listerial effect in the food matrix than in vitro analyses [12,13]. Saraiva et al. [13] evaluated the efficacy of myrtle essential oil against *L. monocytogenes* in sheep milk cheese and reported that the addition of myrtle essential oil showed a lower *L. monocytogenes* (approximately 1–2 log cfu/g) count during the ripening period compared to the control samples. It has also been stated that myrtle essential oil prevented the growth of *L. monocytogenes* in cheeses; however, it had no effect on reducing the bacterial load. Similarly, Dhifi et al. [12] have reported that *Myrtus communis* flower essential oil added to ground beef at the rate of 0.4% and 0.8% had a bacteriostatic effect against *L. monocytogenes* and delayed the growth of bacteria during storage. In the present study, 0.42 and 0.88 log cfu/g reductions were determined in cheese samples coated with sodium alginate nanoemulsion containing 1.0% and 2.0% myrtle essential oil, respectively. Nanoencapsulation of essential oils is an effective approach to increase the physical stability of active compounds and protect them from interaction with food components. The nanoemulsion-based dispersion system can increase the antimicrobial activity of encapsulated essential oils because of its high surface-to-volume ratio and small particle size [34,40].

#### 2.4.2. Change of Physicochemical Properties of Cheese Samples

The changes in the physicochemical properties of Kasar cheese samples during the 24-day storage are shown in Table 4. pH is regarded to be one of the most important factors affecting the texture and flavor of cheese, as it affects the solubility of caseins and the activity of enzymes involved in ripening [41,42]. The pH in all cheese samples ranged from 5.66 ± 0.01 to 5.69 ± 0.01, indicating no significant effect of alginate nanoemulsion coatings with or without myrtle essential oil on the pH of Kasar cheese (Table 4). Silva et al. [43] stated that the alginate coating did not have an effect on the pH of the cheese. On the other hand, the pH increase was observed in all samples on day 6 of the storage period. The slight increase in cheese pH, especially on day 6, can be associated with the release of alkaline compounds during proteolysis [42]. Water activity (a_w_) is the main factor affecting stability in semi-hard cheeses during ripening. The water activity of cheese samples showed relatively high a_w_ values (0.95–0.97) at the beginning of the storage (Table 4). These values remained constant during the storage period for all tested cheese samples without significant differences (*p* > 0.05). At the beginning of the storage, the coated cheese samples showed lower hardness values than the uncoated samples (Table 4). The effect of the coating on the decrease in cheese hardness can be explained by the hydration of the cheese [44]. The hardness values of the cheese samples decreased with increasing essential oil concentration (*p* < 0.05). The hardness of all cheese samples significantly increased during storage (*p* < 0.05). There were no statistical differences among treatments for the hardness parameter at the end of storage (*p* > 0.05). This increase in hardness values could be attributed to the water loss and proteolysis during cheese maturation [32,45]. Similarly, Zhong et al. [46] measured low hardness values at the beginning of the storage in the coated cheeses, but observed that the hardness value was close to that of the control sample at the end of storage. Artiga-Artigas et al. [32] have also reported that the cheese hardness decreased with the coating application; however, this value was not affected by the increase in essential oil concentration. Nanoemulsions are often described as transparent systems due to their smaller droplet size. The transparency of coating solutions is especially important in food applications and affects the acceptability of the product for the consumer. The uncoated control cheese samples had the highest WI value (74.34 ± 0.53) and did not change significantly during storage (*p* > 0.05). On the other hand, the cheese samples coated with alginate nanoemulsion showed lower WI values (68.11 ± 0.82–69.88 ± 0.82) compared to the control (*p* < 0.05). A gradual increase in WI value was observed during storage in all coated samples. Similarly, Artiga-Artigas et al. [32] have reported an increase in WI values during storage in cheeses coated with alginate nanoemulsions containing 1.5% oregano essential oil.

#### 2.4.3. Sensory Evaluation

The effect of different concentrations of myrtle essential oil nanoemulsion coatings on the sensory characteristics of Kasar cheese is shown in Table 5. The coated cheese samples obtained higher sensory scores in terms of color and appearance than those of the control samples because nanoemulsion-based edible coatings offer a glossy appearance to the cheese samples. The odor, flavor, and general acceptability were significantly affected by myrtle essential oil (*p* < 0.05). The highest overall acceptance scores were observed in the samples coated with sodium alginate (7.50) followed by uncoated samples (7.33). The general acceptability, odor, and flavor scores decreased depending on the increase in myrtle essential oil concentration in the alginate nanoemulsion coating. It was found that the acceptability of the Kasar cheese coated with 2% myrtle essential oil was the lowest (5.11) which can be attributed to the taste and the intense smell of myrtle essential oil. However, Kasar cheese samples coated with nanoemulsion edible coating containing myrtle essential oil were considered to be moderately acceptable at all concentrations used in the present study. The results of the sensory analysis revealed that nanoemulsion edible coating incorporated with essential oil can be used without seriously negatively affecting the sensory attributes of the cheese product.

## 3. Materials and Methods

### 3.1. Materials

Fresh Kasar cheese; (protein 24.99%, fat 28.59%, and moisture content 42.22%) was kindly provided by Güneşoğlu Süt A.Ş. (Sakarya, Turkey). Food grade sodium alginate, glycerol, and Tween 80 were purchased from Sigma-Aldrich (St. Louis, MO, USA). Ultrapure water was used for the preparation of the coating solutions. Myrtle essential oil was obtained from BIOMESI Bioagrotechnology R&D (Adana, Turkey). The wild myrtle plants were collected for essential oil analyses from Adana Province in Turkey in November 2021. The dried myrtle leaves were submitted to the hydro-distillation process by using an industrial type of Clevenger apparatus for 4 h. The recovered essential oil was dried over anhydrous sodium sulfate and stored in darkness at 4 °C [47].

### 3.2. Bacterial Strains and Cultural Conditions

*Listeria monocytogenes* ATCC 19111, *L. monocytogenes* ATCC 7644, and *L. monocytogenes* ATCC 13932 were obtained from the American Type Culture Collection ((Manassas, VA, USA). All strains were kept at −18 °C in Tryptic Soy Broth (TSB; Merck, Darmstadt, Germany) containing 15% glycerol. *L. monocytogenes* strains were grown in TSB supplemented with 5 g/L yeast extract (TSBYE; Merck, Darmstadt, Germany) for 24 h at 37 °C.

### 3.3. Analysis of the Composition of Essential Oil

The chemical composition of the myrtle essential oil was determined using gas chromatography–mass spectrometry (GC-MS, Claurus 500; Perkin-Elmer Instruments, Waltham, MA, USA) according to the method of Özoğul et al. [47]. Analysis was performed on an SGE non-polar fused silica capillary column (60 × 0.25 mm, ID-BPX5, 0.25 μm; Perkin Elmer, Shelton, CT, USA) under the following conditions: the oven temperature was programmed at 60–250 °C (4 °C/min), 250 °C (10 min); injector and detector temperature 220 °C; helium was used as carrier gas at a flow rate of 1.5 mL/min; the injection volume was 1μL in splitless mode; the electron ionization mode at 70 eV; the ion source temperature 230 °C; scan mass range 40–550 *m*/*z*, and interface line temperature 250 °C. The compounds of the myrtle essential oil were identified using the NIST-MS and Wiley libraries and compared with the mass spectral data from the literature.

### 3.4. Preparation and Characterization of Nanoemulsion

#### 3.4.1. Nanoemulsion Preparation

The nanoemulsion was formulated using the procedure previously described by Yazgan et al. [48], with minor modifications. The coarse emulsion was prepared from a mixture of myrtle essential oil (10% *w*/*w*), Tween 80 (1% *w*/*w*), and ultrapure water (89% *w*/*w*) and homogenized at 10,000 rpm for 2 min (T25 digital Ultra-Turrax; IKA, Staufen, Germany). The coarse emulsions were sonicated using an ultrasonic processor (VCX 750; Sonics & Materials, Inc., Newtown, CT, USA) for 5 min at 80% power amplitudes with a 13-mm-diameter titanium probe to obtain nanoemulsion.

#### 3.4.2. Particle Size and Zeta Potential

The particle size and zeta potential of coarse emulsions and nanoemulsions were determined by dynamic light scattering (DLS) with a Zetasizer NanoZS laser diffractometer (Malvern Instruments Ltd., Worcestershire, UK) at 25 °C. The mean particle size and ζ-potential of myrtle essential oil nanoemulsion were measured to be 144.6 ± 4.41 nm and, −37.3 ± 0.61, respectively.

#### 3.4.3. Measurement of Minimum Inhibitory and Minimum Bactericidal Concentrations of Myrtle Essential Oil Emulsions and Nanoemulsions

Minimum inhibitory concentrations (MIC) and minimum bactericidal concentrations (MBC) of myrtle essential oil emulsions and nanoemulsions were determined by a broth dilution method in ¼ strength TSBYE amended with 0.15% agar as described by Delaquis et al. [49]. *L. monocytogenes* ATCC 19111, *L. monocytogenes* ATCC 7644, and *L. monocytogenes* ATCC 13932 were cultured individually in TSBYE for 24 h at 37 °C. The essential oil emulsions and nanoemulsions were diluted in a 1:1 ratio with ½ strength TSBYE + 0.30% agar (50 mg/mL). The stock solutions were dispensed into the wells of microtiter plates along with sterile ¼ strength TSBYE + 0.15% agar to achieve concentrations ranging between 0.5 to 50 mg/mL. Each well was then inoculated with 10 µL of culture (final cell concentration ~5 log cfu/mL). MIC was defined as the lowest concentration that prevented visible growth. A loopful of medium from wells without evidence of growth was applied to tryptic soy agar supplemented with 5 g/L yeast extract (TSAYE) which was incubated for 24 h at 37 °C to determine MBC.

#### 3.4.4. Membrane Integrity

The effect of myrtle essential oil nanoemulsion on the membrane integrity of *L. monocytogenes* was performed according to Sugumar et al. [50], with minor modifications. *L. monocytogenes* ATCC 7644 was cultured in TSBYE at 37 °C for 24 h. The cells were harvested by centrifugation at 6000 rpm for 10 min, washed twice, and resuspended in a sterile 0.85% NaCl (final cell concentration ~7 log cfu/mL). The cell suspensions were treated with the MBC of the nanoemulsions at 37 °C for 5, 15, and 30 min. After incubation, the mixture was filtered through 0.22 μm cellulose acetate membrane filter and the absorbance of the treated cell filtrate was read using a UV–visible spectrophotometer (UV-1240; Shimadzu, Kyoto, Japan) at 260 nm (*A*_1_). The leakage of UV absorbance was calculated as (*A*_1_/*A*_0_) × 100. Cell culture treated with Triton X-100 (*A*_0_) or without any treatment was used as positive and negative controls, respectively.

#### 3.4.5. Field Emission Scanning Electron Microscopy (FESEM)

The mode of action of essential oil nanoemulsion on *L. monocytogenes* was confirmed using field emission scanning electron microscopy (FESEM, Quanta 450 FEG; FEI, Hillsboro, OR, USA) as described by Shi et al. [51], with minor modifications. *L. monocytogenes* ATCC 7644 cells were treated with MBC concentration of myrtle essential oil nanoemulsion, and incubated at 37 °C for 15 min. The cells were harvested by centrifugation at 6000 rpm for 10 min, and washed twice with phosphate-buffered saline solution (pH 7.0). The microorganisms were resuspended in water containing 2.5% glutaraldehyde, and then held at 4 °C for 12 h. Subsequently, the cells were gradually dehydrated in water–ethanol series (30%, 50%, 70%, 80%, 90%, and 100% ethanol) for 10 min. Finally, the samples were dried, and gold-sputter coated to prepare for analysis under a FESEM at 20,000× magnification.

### 3.5. Preparation and Characterization of the Nanoemulsion Coating Solution

#### 3.5.1. Preparation of Nanoemulsion-Coating Solution

Sodium alginate (2% *w*/*w*) was dissolved in ultrapure water at 70 °C for 2 h. Coarse emulsions were prepared by mixing the alginate solution (2% *w*/*v*), myrtle essential oil (0.5, 1, and 2% *w*/*w*), glycerol (1.5% *w*/*w*), and Tween 80 (1% *w*/*w*). The mixtures were homogenized at 10,000 rpm for 2 min (T25 digital Ultra-Turrax; IKA, Germany). Then, the coarse emulsions were subjected to ultrasonication using an ultrasonic processor (VCX 750; Sonics & Materials, Inc., Newtown, CT, USA) for 5 min at 80% power amplitudes with a 13-mm-diameter titanium probe.

#### 3.5.2. Particle Size and Zeta Potential

The particle size and zeta potential of the coarse emulsions and nanoemulsions were determined as previously mentioned in Section 3.4.2.

#### 3.5.3. Whiteness Index (WI)

The color values of coarse emulsions and nanoemulsions were determined using a colorimeter (Minolta CM-3600d, Osaka, Japan). The parameters L* (lightness), a* (red-green scale), and b* (yellow-blue scale) were measured. The whiteness index (WI) was calculated using the following equation [32]:

WI = 100 − [(100 − L*)^2^ + (a*^2^ + b*^2^)]^0.5^ (1)

### 3.6. Application of Coatings Solutions on Cheese Samples

The cheese samples were aseptically cut into approximately 10 g pieces (70 mm × 45 mm × 3 mm) and placed onto sterile trays. The cheese samples were randomly divided into five groups (shown in Table 6. All of the groups were individually immersed in alginate nanoemulsion solutions for 2 min and were allowed to drain for 2 min except for the control group. The uncoated samples were immersed in ultrapure water following the same procedure. The cheese samples were placed separately in a sterile bag (Whirl-Pak; Nasco, Wisconsin, USA), and sealed. A total of 300 cheese samples (five treatments × three replicates × four samples × five days) were prepared on different days for physicochemical analyses. Each treatment was stored at 5 °C for 24 days and sampled at days 0, 6, 12, 18, and 24 for the physicochemical analyses. A total of 90 cheese samples (five treatments × three replicates × six panelists) were prepared for the sensory evaluation.

#### 3.6.1. Antibacterial Activity against Inoculated *L. monocytogenes*

The *L. monocytogenes* strains were cultured in TSBYE at 37 °C for 24 h. The sliced cheese pieces were irradiated with ultraviolet light in a laminar airflow cabinet for 15 min to eliminate the background microflora. The cheese samples were tested for the presence of *L. monocytogenes* before the experiments. Each sample was inoculated with 50 µL aliquots of the cocktail of *L. monocytogenes* strains to obtain a final concentration of approximately 4.5 log cfu/g. The inocula were evenly spread over the surface of the cheese samples and allowed to dry for 15 min for the bacterial attachment. The cheese samples were coated as described in Section 3.6, placed separately in a sterile bag (Whirl-Pak; Nasco, WI, USA), and sealed. A total of 120 cheese samples (five treatments × three replicates × eight days) were prepared on different days. Each treatment was stored at 5 °C for 24 days and sampled at regular time-intervals for the analysis. The sample was homogenized with sterile 0.1% (*w*/*v*) peptone water using a stomacher (BagMixer^®^; Interscience, France) for 1 min. Appropriate serially diluted samples were spread onto Oxford Agar plates which were incubated at 37 °C for 24 h. The population of *L. monocytogenes* was expressed as log cfu/g samples.

#### 3.6.2. Physicochemical Properties of Cheese

##### Water Activity, pH, and Color

The water activity (a_w_) was measured twice for each sample using a water activity meter (Aqua Lab Series 3TE; Decagon Devices Inc., Pullman, WA, USA). The surface color of coated and uncoated cheese samples was determined using a colorimeter (PCE-CSM 7; PCE Instruments, UK), and the parameters L*, a*, and b* values were recorded at room temperature. The whiteness index was calculated through Equation (1). Each cheese sample (10 g) was homogenized with distilled water (100 mL) for 1 min using a homogenizer (Ultra-Turrax T25; IKA Labortechnik, Staufen, Germany), and pH was measured by a digital pH-meter (Seven Compact S210; Mettler-Toledo, Switzerland).

##### Hardness Determination

The hardness value of Kasar cheese samples was determined using a texture analyzer (Brookfield AMETEK CT3-4500; Middleboro, MA, USA). Cheese samples were subjected to a compression test at 6–8 °C using a stainless steel, 2-cm-long cylindrical TA39 probe. The test conditions were 10 mm penetration distance, 3 g trigger load, and 5 mm/s speed. The results were expressed in Newton (N).

#### 3.6.3. Sensory Evaluation

The sensory properties of cheese samples were examined after 1 day of storage with the contribution of six semi-trained panelists in cheese-product evaluation at the Department of Food Engineering, Sakarya University. The panelists rated each sample for color, odor, flavor/taste, texture, and overall acceptability, using a 9-point hedonic scale, 1: dislike extremely, 5: neither like nor dislike and 9: like extremely. Cheese samples receiving overall scores of at least 5 were considered acceptable.

### 3.7. Statistical Analysis

Data analysis by ANOVA and Duncan’s multiple range test (level of confidence 95%, *p* < 0.05) was performed using SPSS 20.0 Statistics Software (SPSS, Chicago, IL, USA). All experiments were performed in triplicate and values were expressed as mean ± standard deviation.

## 4. Conclusions

A new nanoemulsion-based edible coating containing myrtle essential oil was developed using ultrasound treatment. Myrtle essential oil showed a potent antibacterial activity against *L. monocytogenes* in in vitro analyses. The antibacterial effect of myrtle essential oil against *L. monocytogenes* increased significantly with ultrasound treatment. The ability of myrtle essential oil nanoemulsion to damage the morphology of *L. monocytogenes* was clearly demonstrated by FESEM. Nanoemulsion coatings containing 0.5% myrtle essential oil showed bacteriostatic activity against target bacteria in cheese, while nanoemulsion coatings containing 1.0% and 2.0% essential oil had bactericidal effects. It was determined that the essential oil concentration is an important factor in inhibiting *L. monocytogenes*. Sodium alginate-based nanoemulsion coatings containing myrtle essential oil did not have a negative effect on the physicochemical properties of cheeses, such as pH, color, and hardness. In addition, the sensory evaluation results indicated that the color and appearance attributes were improved by coating the cheese samples. Coated cheese samples were considered to be acceptable at all myrtle essential oil concentrations. Consequently, nanoemulsion-based alginate edible coatings containing myrtle essential oil may be a promising alternative to synthetic additives to increase food safety.

## Figures and Tables

**Table 1:** Chemical composition of myrtle essential oil.

No	Compound	%
1	Thujene	0.25
2	α-pinene	30.19
3	β-pinene	0.45
4	β-myrcene	0.1
5	α-phellandrene	0.04
6	3-carene	0.23
7	o-cymene	1.14
8	d-limonene	7.51
9	1,8-cineol	38.64
10	β-ocimene	0.1
11	γ-Terpinene	0.09
12	α-Terpinolene	0.19
13	α-ocimene	6.57
14	Terpinen-4-ol	0.2
15	α-terpineol	3.91
16	Myrtenol	1.03
17	β-cis-Ocimene	2.68
18	Verbenene	0.09
19	Thymol	0.1
20	2,4-Thujadiene	0.5
21	Isosylvestrene	2.2
22	Cyclofenchene	0.12
23	(+)-3-Carene	0.69
24	Methyl-eugenol	0.29
25	Caryophyllene	0.55
	Total	97.86

**Table 2:** Minimum inhibitory (MIC) and minimum bactericidal (MBC) concentrations of emulsion and nanoemulsion of myrtle essential oil against *L. monocytogenes* strains.

Strain	Emulsion of Myrtle Essential Oil	
	MIC (mg/mL)	MBC (mg/mL)
ATCC 7644	6.00 ± 0.00 ^b^	8.00 ± 0.00 ^bc^
ATCC 1911	6.00 ± 0.00 ^b^	8.33 ± 0.33 ^b^
ATCC 13932	8.67 ± 0.58 ^a^	14.67 ± 0.58 ^a^
	**Nanoemulsion of Myrtle Essential Oil**	
	**MIC (mg/mL)**	**MBC (mg/mL)**
ATCC 7644	4.00 ± 0.00 ^d^	5.00 ± 0.00 ^d^
ATCC 1911	4.00 ± 0.00 ^d^	5.33 ± 0.58 ^d^
ATCC 13932	4.67 ± 0.58 ^c^	7.33 ± 0.58 ^c^

Results are represented as mean ± standard deviation. Mean values in each column with different lower case letter superscripts are significantly different (*p* < 0.05) (a–d).

**Table 3:** Particle size, zeta potential and whiteness index of alginate nanoemulsion-coating solutions.

Sample	Particle Size (nm)	ζ-Potential (mV)	WI
E_1_	1490 ± 327 ^a^	−15.027 ± 2.76 ^c^	79.22 ± 0.24 ^a^
E_2_	1376 ± 221 ^a^	−11.24 ± 1.02 ^d^	78.30 ± 0.10 ^a^
E_3_	1184 ± 410 ^a^	−15.87 ± 1.36 ^c^	66.23 ± 0.72 ^d^
NE_1_	157 ± 22 ^b^	−38.47 ± 2.48 ^a^	75.65 ± 0.44 ^b^
NE_2_	172 ± 30 ^b^	−32.27 ± 2.94 ^b^	70.76 ± 0.49 ^c^
NE_3_	122.7 ± 1.20 ^b^	−37.37 ± 2.48 ^a^	63,44 ± 0.31 ^e^

Results are represented as mean ± standard deviation. Mean values in each column with different lower case letter superscripts are significantly different (*p* < 0.05) (a–e). E_1_, E_2_, E_3_: alginate coarse emulsion containing 0.5, 1.0, 2.0% myrtle essential oil, respectively. NE_1_, NE_2_, NE_3_: alginate nanoemulsion containing 0.5, 1.0, 2.0% myrtle essential oil, respectively.

**Table 4:** The effect of nanoemulsion coating on the physicochemical properties of the cheese samples.

	Storage Days				
Cheese Samples	0	6	12	18	24
	pH				
C	5.66 ± 0.01 ^Ac^	5.74 ± 0.01 ^Ba^	5.72 ± 0.02 ^Aab^	5.74 ± 0.01 ^Ca^	5.71 ± 0.01 ^Ab^
NA	5.69 ± 0.01 ^Ab^	5.73 ± 0.02 ^Ba^	5.73 ± 0.01 ^Aa^	5.74 ± 0.01 ^Ca^	5.72 ± 0.02 ^Aa^
NE_1_	5.68 ± 0.02 ^Ad^	5.78 ± 0.02 ^Aa^	5.75 ± 0.02 ^Abc^	5.75 ± 0.01 ^ABab^	5.72 ± 0.01 ^Ac^
NE_2_	5.66 ± 0.02 ^Ad^	5.79 ± 0.02 ^Aa^	5.74 ± 0.02 ^Ab^	5.74 ± 0.01 ^BCb^	5.72 ± 0.01 ^Ac^
NE_3_	5.67 ± 0.03 ^Ad^	5.80 ± 0.01 ^Aa^	5.75 ± 0.01 ^Ab^	5.76 ± 0.01 ^Ab^	5.72 ± 0.01 ^Ac^
	a_w_				
C	0.96 ± 0.00 ^Ba^	0.96 ± 0.01 ^Aa^	0.96 ± 0.00 ^Aa^	0.96 ± 0.01 ^Aa^	0.96 ± 0.01 ^Aa^
NA	0.95 ± 0.01 ^BCb^	0.96 ± 0.01 ^Aa^	0.96 ± 0.00 ^Aab^	0.96 ± 0.00 ^ABab^	0.95 ± 0.01 ^Ab^
NE_1_	0.96 ± 0.01 ^BCa^	0.96 ± 0.01 ^Aa^	0.96 ± 0.00 ^Aa^	0.97 ± 0.01 ^Aa^	0.96 ± 0.01 ^Aa^
NE_2_	0.95 ± 0.01 ^Ba^	0.96 ± 0.00 ^Aa^	0.96 ± 0.00 ^Aa^	0.95 ± 0.01 ^BCa^	0.95 ± 0.01 ^Aa^
NE_3_	0.97 ± 0.01 ^Aa^	0.96 ± 0.00 ^Ab^	0.96 ± 0.00 ^Ab^	0.95 ± 0.00 ^Cc^	0.96 ± 0.01 ^Ab^
	WI				
C	74.34 ± 0.53 ^Aa^	74.13 ± 0.31 ^Aa^	74.43 ± 0.24 ^Aa^	74.14 ± 0.35 ^Aa^	74.18 ± 0.62 ^Aa^
NA	68.41 ± 0.57 ^CDc^	68.54 ± 0.73 ^Dc^	71.04 ± 0.65 ^Bb^	70.75 ± 0.64 ^Cb^	71.64 ± 0.46 ^Ba^
NE_1_	69.88 ± 0.72 ^Bc^	69.90 ± 0.67 ^BCc^	69.35 ± 0.60 ^Cc^	70.66 ± 0.69 ^Cb^	71.58 ± 0.83 ^Ba^
NE_2_	68.11 ± 0.82 ^Dd^	69.60 ± 0.57 ^Cc^	69.10 ± 0.77 ^Cc^	70.96 ± 0.49 ^BCb^	71.82 ± 0.52 ^Ba^
NE_3_	68.88 ± 0.77 ^Cc^	70.30 ± 0.73 ^Bb^	71.02 ± 0.53 ^Ba^	71.39 ± 0.59 ^Ba^	71.54 ± 0.56 ^Ba^
	Hardness (N)				
C	2.18 ± 0.02 ^Ac^	2.88 ± 0.27 ^ABab^	2.98 ± 0.17 ^BCab^	3.08 ± 0.20 ^ABa^	2.72 ± 0.15 ^Ab^
NA	2.11 ± 0.03 ^Ad^	2.80 ± 0.04 ^Bbc^	2.61 ± 0.14 ^Cc^	2.99 ± 0.16 ^ABab^	3.07 ± 0.15 ^Aa^
NE_1_	2.08 ± 0.04 ^Ad^	2.57 ± 0.13 ^Bc^	3.69 ± 0.32 ^Aa^	3.06 ± 0.34 ^ABb^	2.81 ± 0.32 ^Abc^
NE_2_	1.86 ± 0.12 ^Bb^	2.69 ± 0.09 ^Ba^	2.70 ± 0.21 ^Ca^	2.69 ± 0.44 ^Ba^	2.95 ± 0.11 ^Aa^
NE_3_	1.74 ± 0.03 ^Cc^	3.17 ± 0.21 ^Aa^	3.13 ± 0.16 ^Ba^	3.40 ± 0.22 ^Aa^	2.82 ± 0.10 ^Ab^

Results are represented as mean ± standard deviation. Mean values in each row with different lower case letter superscripts are significantly different (*p* < 0.05) (a–d). Mean values in each column with different upper case letter superscripts are significantly different (*p* < 0.05). C: Control, uncoated cheese; NA: samples coated with alginate nanoemulsion coating; NE_1_, NE_2_, NE_3_: samples coated with alginate nanoemulsion coating containing 0.5, 1.0, or 2.0% myrtle essential oil, respectively.

**Table 5:** The effect of nanoemulsion coating on the sensory properties of the cheese samples.

Samples	Appearance	Odor	Color	Flavor	General Acceptability
C	7.17 ± 1.20 ^b^	7.39 ± 1.04 ^a^	6.89 ± 1.32 ^b^	7.67 ± 0.97 ^a^	7.33 ± 1.03 ^a^
NA	7.72 ± 1.13 ^ab^	7.22 ±0.88 ^a^	7.89 ± 0.90 ^a^	7.22 ± 1.06 ^a^	7.50 ± 0.86 ^a^
NE_1_	7.67 ± 0.49 ^ab^	6.94 ± 1.16 ^ab^	7.44 ± 1.04 ^ab^	6.06 ± 1.83 ^b^	6.28 ± 1.64 ^b^
NE_2_	7.89 ± 0.47 ^a^	7.00 ± 1.37 ^ab^	7.89 ± 0.47 ^a^	5.72 ± 1.90 ^bc^	6.00 ± 1.91 ^bc^
NE_3_	7.78 ± 1.00 ^ab^	6.28 ± 1.60 ^b^	7.72 ± 1.27 ^a^	5.00 ± 1.50 ^c^	5.11 ± 1.68 ^c^

Results are represented as mean ± standard deviation. Mean values in each column with different lower case letter superscripts are significantly different (*p* < 0.05) (a–c). C: Control, uncoated cheese; NA: samples coated with ultrasound-treated sodium alginate solution without the incorporation of myrtle essential oil; NE_1_, NE_2_, NE_3_: samples coated with ultrasound-treated sodium alginate solution with the incorporation of 0.5, 1.0, or 2.0% myrtle essential oil, respectively.

**Table 6:** List of treatments in the present study.

No	Treatment	Description
1	C	Control, uncoated cheese
2	NA	Alginate nanoemulsion coating
3	NE_1_	Alginate nanoemulsion coating containing 0.5% myrtle essential oil
4	NE_2_	Alginate nanoemulsion coating containing 1.0% myrtle essential oil
5	NE_3_	Alginate nanoemulsion coating containing 2.0% myrtle essential oil

## Data Availability

Not applicable.
